# Pharmacologic Mechanisms of *Portulaca oleracea* L. in the Treatment of Musculoskeletal Disorders: A Mechanistic Review

**DOI:** 10.1002/fsn3.70920

**Published:** 2025-09-22

**Authors:** Yanxin Li, Yan Wang, Xiaotian Feng, Qiyu Wang, Hongwei Cui, Tiancheng Ma, Yujing Chen, Jiajun Liu, Jianxiong Ma

**Affiliations:** ^1^ Tianjin Hospital Tianjin University Tianjin China; ^2^ Tianjin Orthopaedic Institute of Integrated Traditional Chinese and Western Medicine Tianjin China; ^3^ Tianjin Key Laboratory of Orthopedic Biomechanics and Medical Engineering Tianjin China

**Keywords:** herbal medicine, mechanism, musculoskeletal disorders, *Portulaca oleracea* L.

## Abstract

*Portulaca oleracea* L. is a plant with nutritional and medicinal value. It is known for its rich bioactive compounds and its potential to benefit musculoskeletal disorders. Recent studies have explored its pharmacological mechanisms within this context. This study aims to summarize the pharmacological effects and molecular mechanisms of *Portulaca oleracea* L. in treating musculoskeletal disorders to support its therapeutic use. We systematically searched Web of Science, PubMed, and Scopus. We organized our work according to the PRISMA statement and screened studies for review according to predetermined selection criteria. After screening, we identified 69 relevant studies, including 9 clinical trials and 60 experimental studies. Our findings suggest that *Portulaca oleracea* L. primarily operates through key signaling pathways and reduces cytokines such as tumor necrosis factor‐α, interleukin‐6, and interleukin‐1β. These effects could alleviate inflammation and oxidative stress, prevent bone degeneration, and indicate its potential as a treatment for musculoskeletal disorders. Notably, the involvement of the PI3K/AKT, Nrf2/ARE, MAPK, and RANKL/OPG signaling pathways highlights the herb's promise for future clinical application.

## Graphical abstract

We conducted a comprehensive search of publicly available databases. The main focus is to investigate the pharmacological effects and mechanisms of *Portulaca oleracea* L. in the treatment of musculoskeletal disorders. This study aims to provide a reference for further research into the active components of this plant.

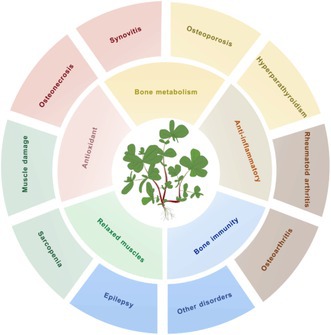

## 1 Introduction

The musculoskeletal system comprises muscles, bones, and connective tissues that work together to support movement and overall well‐being (Safiri et al. 2021). The global population aged 60 and above exceeds one billion, accounting for 13.7% of the total population. The rising global aging population notably affects public health, particularly in the prevalence of musculoskeletal disorders (Seya et al. 2011). This demographic shift has led to a rise in the incidence and disability rates of various musculoskeletal disorders, including sarcopenia, osteoarthritis, osteoporosis, rheumatoid arthritis, and so on. Current estimates indicate that approximately 200 million individuals suffer from osteoporosis worldwide (Steinhoff and Reiner 2024). Additionally, the incidence of osteoarthritis among those aged 60 and older has escalated to 37%, while the global prevalence of sarcopenia is around 10%. Recent studies suggest that the total number of individuals with musculoskeletal disorders has reached 1.3 billion, leading to a disability rate of 10.67% (Onizuka and Onizuka 2024). The high prevalence of these diseases has resulted in a significant decline in patients' quality of life, with common clinical manifestations including chronic pain, functional impairment, and reduced mobility.

Although various treatment options for musculoskeletal disorders are available, including medications, surgery, and biologics. However, the associated financial burden and side effects affect patients and families significantly. In 2016, the cost of musculoskeletal disorders in the United States reached approximately $380.9 billion, making it the highest healthcare expenditure category (Dieleman et al. 2020). Nonsteroidal anti‐inflammatory drugs are commonly used for mild pain relief but can cause gastrointestinal ulcers, gastric bleeding, and renal issues with prolonged use. Opioid analgesics are effective but pose risks of sedation and dependence. In low‐ and middle‐income countries, more than 550 million individuals face inadequate pain management due to limited access to opioid analgesics (Scholten et al. 2020). Furthermore, the consumption of tramadol varies widely, with a 20‐fold difference between high‐income and low‐income nations (Jayawardana et al. 2021). Surgical options can alleviate pain but are costly and carry risks such as infection and the need for revision surgeries. The average cost of total knee replacement is about $19,568 in the United States, while it is approximately $3457 in countries like India. This disparity emphasizes the urgent need for affordable and effective treatments with minimal side effects (Gandhi et al. 2023).

**FIGURE 1:**
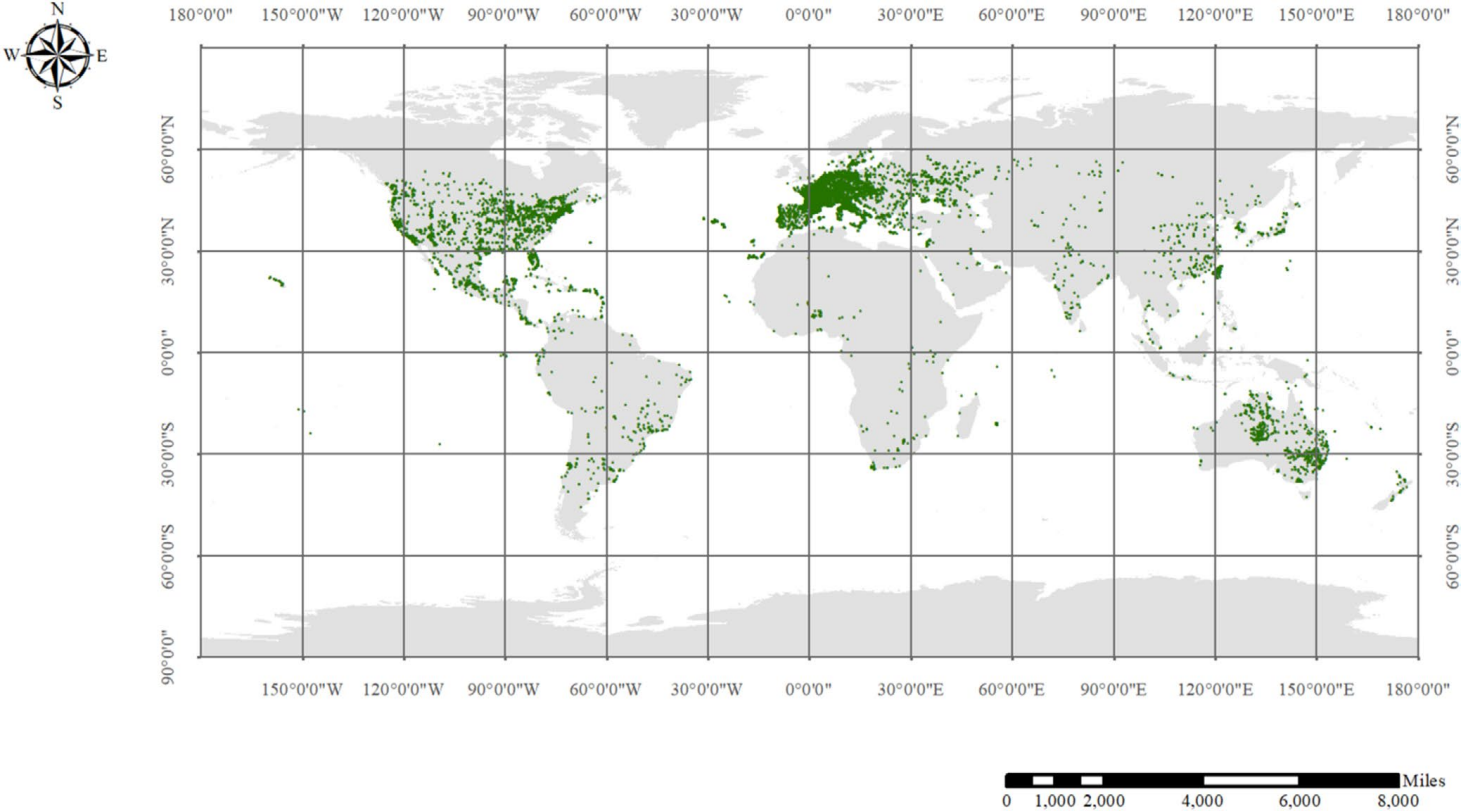
Global species distribution map of POL (Global biodiversity information facility).

*Portulaca oleracea* L. (POL), known as purslane, is an annual herb in the Portulacaceae family. It is native to India and the Middle East but is now found in temperate, subtropical, and tropical regions worldwide (Figure 1). The plant typically grows to about 30 cm in length and is characterized by its succulent. Its oval or oblong green leaves and five yellow petals characterize its appearance (D'Imperio et al. 2017). POL is rich in minerals such as potassium, calcium, and magnesium. It serves both culinary and medicinal purposes. Additionally, the plant contains bioactive compounds, including polysaccharides, organic acids, flavonoids, and alkaloids (Li et al. 2024). Studies show that purslane enhances antioxidant activity, reduces inflammation, supports bone metabolism, and improves muscle function (Sourani et al. 2023). As a traditional medicinal plant, POL's rich nutritional and active components show significant promise for treating musculoskeletal disorders.

## 2 Methods and Results of the Literature Search

### 2.1 Methods

We searched and included literature based on PRISMA reports. A total of three databases were searched: PubMed, Web of Science, and Scopus. The search strategy included subject terms combined with arbitrary fields. The keywords included: “ *Portulaca oleracea* ,” “ *Portulaca oleracea* L.,” “ *Portulaca oleracea* extract,” “ *Portulaca oleracea* seed extract,” “ *Portulaca oleracea* leaf extract,” “muscle,” “bone,” “myocyte,” “myofibroblast,” “osteocyte,” “osteoblast,” “osteoclast,” “sarcopenia,” “satellite cell,” “osteoarthrosis,” “osteonecrosis,” “osteoporosis,” “joint,” “synovial membrane,” “rheumatoid arthritis,” “cartilage,” and others.

**FIGURE 2:**
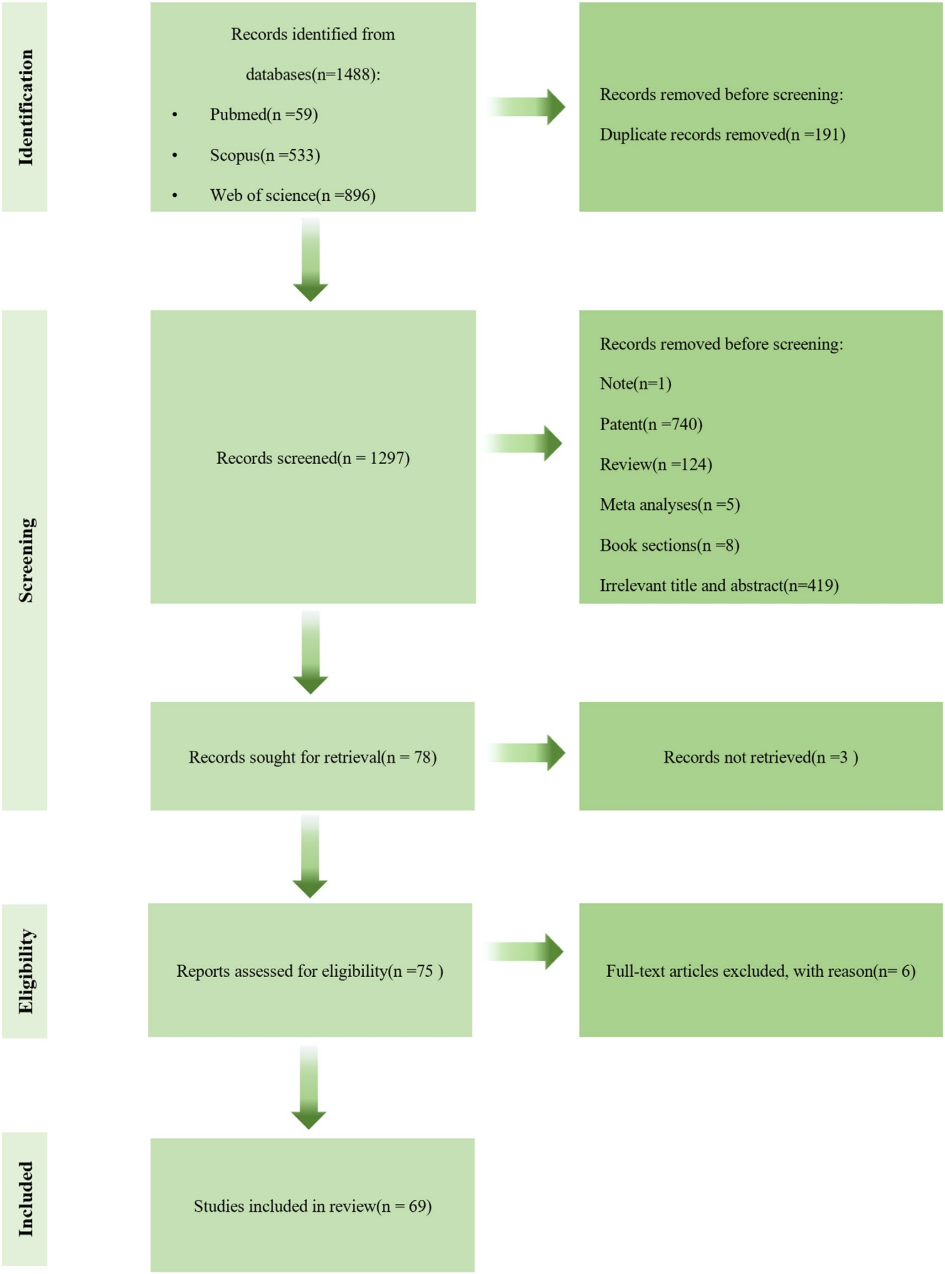
Flowchart of literature search and screen.

The inclusion criteria for the literature review were studies focused on POL and its active ingredients in animal, cellular, and clinical trials Studies included should be related to the musculoskeletal system. The search deadline is January 6, 2025 Meta‐analysis and review articles were excluded, as were articles for which the full text was unavailable. The process of conducting the literature search and screening process is described in Figure 2.

## 3 Musculoskeletal System Overview

The musculoskeletal system provides essential support for movement and mechanical stability and protects internal organs when the body is subjected to impact. Additionally, this system plays a role in multiple physiological processes such as regulating calcium–phosphorus metabolism, immune function, and energy metabolism, all mediated by a complex network of signaling pathways (Dong et al. 2024). The musculoskeletal system functions through the interplay of the extracellular matrix, hormones, and biochemical signals. The close coordination of muscles and bones is essential for sustaining physiological functions and overall health (Lin et al. 2024).

### 3.1 Physiological Functions of Muscles and Bones

Bones provide structure and regulate mineral metabolism, especially calcium and phosphorus. They consist of osteoprogenitor cells, osteoblasts, osteoclasts, and osteocytes, which work together to maintain homeostasis. Osteoprogenitor cells activate in response to stimuli, becoming osteoblasts to support bone stability (Sui et al. 2024). Osteoblasts create new bone by secreting type I collagen, proteoglycans, and osteocalcin. These substances form the bone matrix, which mineralizes through calcium salt deposition. Osteoclasts dissolve mineralized bone, enabling resorption and remodeling. Osteocytes are the most abundant bone cells and reside in the mineralized matrix. They exhibit mechanosensory capabilities that modulate the activity of osteoblasts and osteoclasts in response to mechanical stimuli. Thus, bone remodeling and formation are ongoing processes influenced by interactions among these cells (Yin et al. 2024).

Skeletal muscles connect to bones via tendons and comprise muscle cells, connective tissues, blood vessels, and nerves. They produce movement through the contraction of muscle cells, which contain essential proteins like actin and myosin. Motor neurons regulate these contractions at the neuromuscular junction. Meanwhile, blood vessels supply oxygen and nutrients to meet the metabolic demands of the muscle tissue (Bonewald et al. 2013). Muscle growth and repair depend on myogenic cells, which are located on the surface of mature muscle fibers. These cells activate in response to muscle damage or external stimuli, leading to their proliferation and differentiation. They eventually fuse to form new muscle fibers, helping repair damage and promote muscle hypertrophy. This process is regulated by growth factors and signaling pathways, such as insulin‐like growth factor 1 (IGF‐1) and transforming growth factor‐beta (TGF‐β) (Dallas et al. 2013).

### 3.2 Muscle–Bone Interactions

Muscle and bone tissues, derived from the mesoderm, are essential for body structure and growth. Muscle tension facilitates movement, while bones serve as attachment points. The growth plate regulates rapid bone growth before, coordinating with muscle development (Dong et al. 2024). The size and strength of muscles progressively increase during bone growth, providing support and protection for the skeleton (Kaji 2024).

Muscle and bone coordination relies on both external physical loads and internal signaling regulation. These tissues are biomechanically interdependent and adapt to environmental changes through various signaling pathways. Recent studies highlight the IGF/IGF1R signaling pathway's critical role in muscle–bone communication. IGF‐1 binds to the IGF1R receptor, activating the phosphatidylinositol‐3‐kinase/protein kinase B (PI3K/AKT) pathway, which enhances muscle protein synthesis and promotes muscle repair via the mammalian target of rapamycin (mTOR) pathway (Lin et al. 2024). As a bone‐derived factor, osteocalcin regulates muscle metabolism by binding to the GPRC6A receptor on muscle cells. Additionally, IGF‐1 stimulates osteocalcin secretion, enhancing the interplay between bones and muscles for optimal functional balance (Sharir et al. 2011). Moreover, IGF‐1 influences bone resorption by regulating osteoclast activity, further modulating bone remodeling. Through interactions with receptor activator of nuclear factor kappa‐B ligand (RANKL), IGF‐1 promotes bone formation while simultaneously regulating bone resorption to maintain skeletal health. Additionally, the PI3K/AKT pathway, which regulates cell growth, proliferation, and metabolism, plays an essential role in muscle–bone interactions (Sui et al. 2024). After muscle injury, the PI3K/AKT pathway promotes protein synthesis and enhances muscle cell growth, facilitating muscle repair and functional recovery. Activation of the PI3K/AKT pathway boosts protein synthesis and inhibits its catabolic processes. Furthermore, this pathway promotes osteoblast function by upregulating bone transcription factors such as Runt‐related transcription factor 2 (Runx2) and Osterix.

Meanwhile, muscle–bone interactions are also influenced by other signaling pathways, with antioxidant stress responses. The nuclear factor‐erythroid 2‐related factor 2 (Nrf2) pathway, essential to cellular antioxidant responses, helps maintain homeostasis in both bones and muscles. In aging muscle cells, oxidative stress activates the Nrf2 pathway, which regulates the antioxidant response element (ARE) to initiate the transcription of related antioxidant genes (Yin et al. 2024). This reduces the effects of free radicals and oxidative damage, promoting muscle repair and regeneration. Similarly, bone tissue utilizes the Nrf2 pathway to mitigate oxidative damage, balancing bone formation and resorption to maintain bone density. During bone mineralization and remodeling, Nrf2 enhances antioxidant activity, regulating osteoclast activity and reducing bone loss (Bonewald et al. 2013).

**FIGURE 3:**
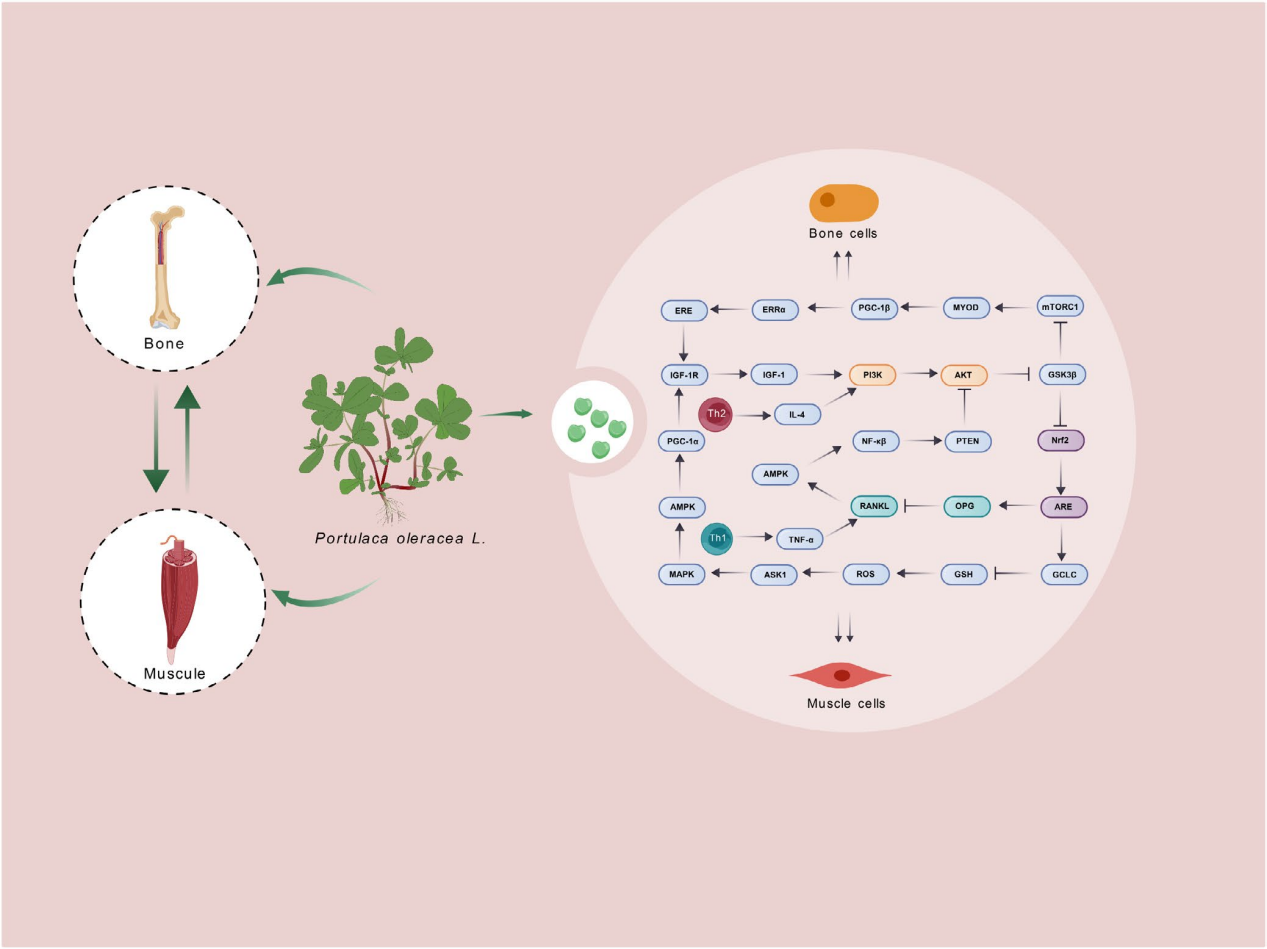
Mechanism diagram for POL in treating musculoskeletal disorders.

Recent research highlights the role of inflammation in muscle–bone interactions. Nuclear factor kappa‐B (NF‐κβ) is a crucial transcription factor associated with inflammation. It plays a significant role in the development and progression of musculoskeletal disorders (Dallas et al. 2013). In osteoporosis and sarcopenia, NF‐κβ activation increases bone resorption and leads to bone density loss. It also plays a key role in muscle atrophy during chronic inflammation by promoting muscle protein degradation. This results in a decline in muscle mass and function. Lastly, we summarized the role of POL in the aforementioned mechanism of muscle–bone crosstalk (Figure 3).

## 4 POL and Its Active Compounds in the Treatment of Musculoskeletal Disorders

### 4.1 Antioxidant Function

Oxidative stress arises from an imbalance between oxidative processes and antioxidant defenses. It occurs mainly due to excess reactive oxygen species (ROS). Oxidative stress is a significant pathogenic factor in many musculoskeletal disorders. It inhibits mesenchymal stem cell differentiation into osteoblasts. Additionally, it causes cell cycle arrest. ROS can also break down fatty acids into lipid peroxides. This degradation damages cellular membranes and impairs their function. Consequently, oxidative stress leads to apoptosis in bone and muscle cells. It also harms the extracellular matrix and activates inflammatory pathways. These effects contribute to osteopenia, muscle atrophy, and joint degeneration.

Studies have demonstrated that POL exhibits significant antioxidant effects by activating multiple signaling pathways, forming a multi‐layered antioxidant regulatory network. These actions significantly alleviate oxidative stress, reduce inflammation, and protect cells and tissues (Table 1). Nrf2 is a key element in antioxidant defense mechanisms and it typically binds to Keap1 for degradation. In response to oxidative stress, Nrf2 is released when ROS induces the oxidation of Keap1's sulfhydryl groups. Nrf2 enters the nucleus and binds to ARE, enhancing the expression of antioxidant enzymes like superoxide dismutase (SOD), glutathione peroxidase (GPx), and catalase (CAT). Research has shown that polyphenols and flavonoids in POL significantly enhance Nrf2 activity. In animal experiments, POL polysaccharides significantly increased SOD and GPx activity while reducing malondialdehyde (MDA) levels by more than 25% (Chen et al. 2014; Kazemi et al. 2018; Lou et al. 2011). According to research, adding POL to the diets of broiler chickens could improve growth performance, increase muscle antioxidant stability and moisture content, and not affect muscle pH adversely (Safari et al. 2016). In food preservation studies, POL extract significantly inhibited lipid and protein oxidation in refrigerated meats, extending shelf‐life and improving food quality (Fan et al. 2019). Habibian et al. (2017) supplemented POL powder to the diet of male broiler chicks and observed a significant reduction in the MDA concentration in the plasma and liver tissue of these chicks. Concurrently, SOD, CAT, and GPx activity increased accordingly, indicating that POL possesses a significant antioxidant effect. In addition, food research further indicates that the addition of POL extracts would effectively inhibit the reduction in gel cooking loss rate of pork myofibrillar proteins. This could improve the gelation and emulsification properties of meat (Shi et al. 2024).

**TABLE 1:** Studies on POL in antioxidant properties.

Compound	Dose	Exposure condition	Model	Results	References
Polysaccharides from purslane	100, 200, 400 mg/kg	28 days, oral	Forced swimming mice	Increased endurance, reduced oxidative stress (lower MDA), and increased antioxidant enzyme activities (SOD, GPx, CAT)	Chen et al. (2014)
Purslane hydroextract (PHE) and zinc	300 and 600 mg/kg PHE + 100 mg/kg zinc	44 days, broiler chickens under heat stress	Broiler chickens	Improved body weight, reduced oxidative stress (MDA, ASAT, LDH), enhanced antioxidant enzymes (SOD, TAC), increased immunity (SRBC, Newcastle)	Kazemi et al. (2018)
Polysaccharides from purslane	100, 200, 400 mg/kg	30 days, oral	Male Sprague–Dawley rats under acute exercise	Improved exercise tolerance (blood lactate), reduced oxidative stress markers (MDA, SOD), and increased antioxidant levels	Lou et al. (2011)
Dried purslane powder	0%, 2.5%, 5%, and 7.5% per 100 g	60 days, oral	Chicken thigh muscle	Feed intake and daily weight gain increased, feed conversion rate improved, and lipid oxidation degree decreased while water content increased	Safari et al. (2016)
POL extract	0.25%, 0.50%, and 1.0% (10 mL)	9 days, refrigerated pork tenderloin	Pork meat samples	Dose‐dependent reduction in lipid oxidation (SOD, GPx), microbial growth, and improved meat quality.	Fan et al. (2019)
Purslane powder	1.5 and 3 g/kg	49 days diet	Broiler chickens with triiodothyronine‐induced ascites	Improved oxidative status (lower MDA), increased antioxidant enzyme activity (SOD, GPx, CAT), and reduced ascites mortality	Habibian et al. (2017)
POL extract (PE)	0, 2, 4, 8 mg/mL PE	Fenton oxidation model, 4°C, 12 h	Pork myofibrillar proteins	Reduced cooking loss, improved water‐holding capacity, increased hardness and elasticity, and enhanced emulsification stability	Shi et al. (2024)
Purslane extract	100 and 200 mg/kg	14 days, IP injection	Rats with chronic constriction injury	Alleviated neuropathic pain, reduced inflammatory cytokines (TNF‐α, IL‐1β), and oxidative damage markers (MDA)	Forouzanfar et al. (2019)
Purslane supplementation	500 mg/capsule, 2 capsules/day	12 weeks, double‐blind clinical trial	Patients with rheumatoid arthritis	Improved clinical outcomes, reduced inflammation (hs‐CRP, TNF‐α, ESR), increased antioxidants (SOD, TAC)	Karimi et al. (2024)
Purslane supplementation	1000 mg/day	10 days, post‐exercise	Female runners, HIIE treadmill	Reduced oxidative stress (9‐HODE, 13‐HODE), inflammation (IL‐17, IL‐10, TNF‐α), and muscle damage biomarkers (LDH) after high‐intensity exercise	Abad et al. (2021)
Purslane seed supplementation	1000 mg/day (500 mg twice per day)	10 days, post‐exercise	Female runners, HIIE treadmill	Reduced oxidative stress markers (9‐HODE, 13‐HODE), inflammation (IL‐17, TNF‐α), and muscle damage (LDH)	Zare et al. (2020)
Purslane extract	0.1%, 0.3%, and 0.5% (10 mL)	12 days, chilled male Ira rabbit meat patties	Male Ira rabbit meat patties	Reduced lipid and protein oxidation, with strong antioxidant properties for meat preservation	Wang et al. (2021)

The NF‐κβ pathway serves as a crucial link between inflammation and oxidative stress. Activation occurs via ROS through Iκβ kinase (IKK), leading to Iκβ degradation and NF‐κβ release into the nucleus. This translocation leads to the expression of pro‐inflammatory cytokines, including tumor necrosis factor‐α (TNF‐α), interleukin (IL)‐1β, and IL‐6, which further stimulate ROS production, perpetuating a vicious cycle. POL effectively inhibits IKK activity, thereby diminishing NF‐κβ activation and lowering cytokine levels. In a chronic neuroinflammation model, POL extract markedly reduced TNF‐α, IL‐1β levels, and MDA generation (Forouzanfar et al. 2019). Studies on rheumatoid arthritis (RA) patients have shown that supplementation with POL extract significantly reduced C‐reactive protein (CRP) levels and enhanced overall antioxidant capacity (Karimi et al. 2024). These findings suggest that POL regulates the interaction between inflammation and oxidative stress via the NF‐κβ pathway, demonstrating its multiple benefits in both anti‐inflammatory actions and food quality improvement.

The PI3K/AKT pathway regulates antioxidant enzyme gene expression through FOXO transcription factors, maintaining cellular viability. PI3K would produce PIP3 upon activation of this pathway, which further activates AKT and promotes cell metabolism and repair. Polysaccharides and polyphenols in POL significantly enhance PI3K/AKT activity, notably lowering ROS and lactate dehydrogenase (LDH) levels. In an exercise‐induced oxidative stress model, POL extract reduced lipid oxidation markers such as 9‐HODE and 13‐HODE and decreased LDH release, thereby alleviating muscle damage (Abad et al. 2021; Zare et al. 2020). The PI3K/AKT pathway's role extends to food preservation, where POL extract improved protein antioxidant capacity, enhancing food shelf‐life and processing characteristics, supporting its application in the food industry (Wang et al. 2021).

### 4.2 Anti‐Inflammatory Function

Chronic inflammation leads to the degradation of bone and muscle tissues and decreased repair capabilities. Inflammatory stimuli activate macrophages and monocytes in synovial tissue, increasing pro‐inflammatory cytokines like IL‐1β and TNF‐α. These cytokines activate the mitogen‐activated protein kinase (MAPK) signaling pathway in chondrocytes, leading to the upregulation of matrix metalloproteinases and the eventual degradation of articular cartilage. The NF‐κβ pathway is key to the inflammatory process. POL and its active ingredients have a certain effect in treating inflammatory reactions in the musculoskeletal system (Table 2). Cannavacciuolo et al. (2022) determined that POL extracts are abundant in polar lipids that effectively inhibit the NF‐κβ pathway and activate Nrf2 and peroxisome proliferator‐activated receptor γ (PPAR‐γ). In another experiment, HM‐chromanone, which is extracted from POL, was found to reduce levels of inflammatory factors and chemokines in a dose‐dependent manner (Kang et al. 2022). It was also found to inhibit the activation of the MAPK and NF‐κβ pathways and improve insulin‐stimulated glucose uptake. Ahmadi et al. (2019) revealed that POL extracts exhibit significantly stronger analgesic and anti‐inflammatory properties than ibuprofen in mouse studies. Gas chromatography–mass spectrometry analysis revealed that flavonoids, anthraquinones, and terpenoids may play a key role in these effects. Another study also found that POL petroleum ether extracts are rich in flavonoids, terpenoids, and alkaloids, which significantly reduce acetic acid‐induced abdominal contractions in mice and alleviate paw edema in rats (Jagan Rao et al. 2012). Previous studies have shown through clinical and animal experiments that TNF‐α levels significantly decrease in the POL group after intervention (He et al. 2021; Karimi et al. 2024). This factor is a key activator of the MAPK pathway. Of course, POL inhibited the nuclear translocation of p65 and downregulated the expression of cyclooxygenase‐2 (COX‐2) and inducible nitric oxide synthase (iNOS). This reduced production of prostaglandin E2 and nitric oxide significantly alleviates the inflammatory response, protecting joints and surrounding tissues. In addition, p65 as a major transcriptionally active subunit of NF‐κβ could drive inflammatory factors such as TNF‐α for activating the MAPK pathway (Miao et al. 2019).

**TABLE 2:** Studies on POL in anti‐inflammatory effects.

Compound	Dose	Exposure condition	Model	Results	References
Purslane extract	1200 mg/day	From 72 h before training until 48 h after, take orally	Healthy non‐athlete students took part in a 35‐min, one‐session bench‐stepping exercise	Reduced serum LDH, Cortisol (24–48 h post‐exercise), CK (48 h post‐exercise), IgA (immediately post to 48 h post‐exercise); lower pain perception (48 h post‐exercise); improved right knee range of motion and maximal isometric force (48 h post‐exercise)	Meamarbashi and Abedini (2011)
Ethanol extracts from POL	50, 100, 200 mg/kg。	Intragastric administration for 7 days	Zymosan‐induced joint inflammation mice	Inhibited articular mechanical hyperalgesia and edema; ameliorated recruitment of mononuclear neutrophils and leukocytes; regulated Nrf2‐related proteins; inhibited NLRP3 inflammasome‐related genes (TNF‐α, IL‐1β, IL‐10) and NF‐κβ activation	He et al. (2021)
Water extracts from POL; ethanol extracts from POL	For antioxidant activity detection: 250, 500, 1000 μg/mL; for anti‐inflammatory activity detection: 500, 1000, 2000, 4000 μg/mL	Cultivate for 24 h	1 μg/mL LPS stimulated raw 264.7 macrophages for 24 h	Both extracts decreased NO and ROS production; ethanol extract reduced pro‐inflammatory cytokines (TNF‐α, IL‐1, IL‐6) expression more than water extract	Kim et al. (2018)
Purslane ethanolic extract	300 mg/kg body weight (high dose); 150 mg/kg body weight (low dose)	Intragastric administration for 8 weeks	Rats received complete Freund's adjuvant (5 mg/mL suspension) with high‐fat diet and 1 mL tween 80 for an 8‐week period	Significant improvement in plasma biomarkers (total cholesterol, triglycerides, HDL‐cholesterol, LDL‐cholesterol, CRP, ESR, RF, Anti‐CCP) in high‐dose group compared to induced arthritis‐high‐fat‐diet group	Elharrif et al. (2024)
(E)‐5‐hydroxy‐7‐methoxy‐3‐(2′‐hydroxybenzyl)‐4‐chromanone (HM‐chromanone) isolated from POL	10, 20, 30, 50 μM	Cultivate for 24 h	1 μg/mL LPS stimulated raw 264.7 macrophages for 24 h	Inhibited production of intracellular ROS, superoxide anion, lipid peroxide; enhanced activity of antioxidant enzymes (SOD, catalase, GSH‐px); inhibited NO and PGE2 production by downregulating iNOS and COX‐2; suppressed LPS‐induced expression of Iκβ, NF‐κβ, TNF‐α, IL‐1β, IL‐6	Kang et al. (2021)
Petroleum ether extract of POL	50, 100, 200 mg/kg	Single dose	Intraperitoneal injection of acetic acid, formaldehyde, and carrageenan in mice	Significantly exerted analgesic effects (reducing pain responses and prolonging pain tolerance) and anti‐inflammatory effects (reducing inflammatory edema)	Jagan Rao et al. (2012)
Hydroalcoholic extract of POL; aqueous phase fraction of POL	Hydroalcoholic extract of POL (80 mg/kg/day); aqueous phase fraction of POL (30, 60, 120 mg/kg/day)	Intragastric administration for 21 days	Mice were exposed to magnetic pressure for 12 h and then released for 12 h；HUVEC cells and HaCaT cells	Significantly increased cell viability, migration rate, and tube formation, reduced muscle iron concentration, decreased inflammatory infiltration (MMP‐9), and increased collagen deposition	Guo et al. (2022)
Purslane tea	7.5 g/L	4 days, oral	Rats with collagen induced arthritis 21 days	Inhibited joint erosion and pannus formation in synovial joints; prevented synovial joint damage	Asmaliani et al. (2024)
(E)‐5‐hydroxy‐7‐methoxy‐3‐(2′‐hydroxybenzyl)‐4‐chromanone (HM‐chromanone) isolated from POL	15, 25, 50 μM	Cultivate for 24 h	3 T3‐L1 adipocytes were treated with TNF‐α (50 ng/mL) for 24 h, and raw 264.7 macrophages were treated with LPS (1 μg/mL) for 24 h	Inhibited production of inflammatory cytokines (TNF‐α, IL‐6, IL‐1β, MCP‐1); decreased JNK phosphorylation; blocked IKK, Iκβα, NF‐κβp65 activation; inhibited IRS‐1 serine 307 phosphorylation; increased IRS‐1 tyrosine 612 phosphorylation	Kang et al. (2022)
Purslane lipid enriched fractions	5, 10, and 20 μg/mL	NF‐κβ inhibition experiment (1 h), Nrf2 activation experiment (16 h), PPAR‐γ activation experiment (16 h)	HEK293 cells and HepG2 ARE‐luc cells were cultured with 2 ng/mL TNF‐α for 4 h	Inhibited TNF‐α‐stimulated NF‐kB pathway; activated PPAR‐ɣ and Nrf2 transcription factors	Cannavacciuolo et al. (2022)
Purslane supplementation	500 mg/capsule, 2 capsules/day	12 weeks, oral	Patients with rheumatoid arthritis	Improved clinical outcomes, reduced inflammation (hs‐CRP, TNF‐α, ESR), increased antioxidants (SOD, TAC)	Karimi et al. (2024)
POL extract	0, 100, 200 and 400 μg/mL)	Cultivate for 4 h	LPS stimulated raw 264.7 macrophages	Inhibits NF‐κβ (P65, p‐P65, Iκβ‐α, p‐Iκβ‐α) and MAPK (p‐MEK, p‐P38, p‐JNK) pathways, reducing the expression of inflammatory factors (NO, iNOS, COX‐2)	Miao et al. (2019)
POL extract	200 and 400 mg/kg	30 min, intraperitoneal injection	Eighty‐eight adult male mice were immersed in water at 52°C and injected with formaldehyde	Pain tolerance time and foot swelling were significantly improved	Ahmadi et al. (2019)

The excessive release of inflammatory cytokines can further amplify inflammation via the MAPK pathway. The MAPK family includes extracellular signal‐regulated kinase (ERK), c‐Jun N‐terminal kinase (JNK), and p38 kinase. When these kinases are phosphorylated, they enhance the release of inflammatory cytokines, thereby escalating the inflammatory response. MAPK pathway activation could upregulate matrix metalloproteinase (MMP)‐9 expression. However, POL extract could inhibit excessive inflammation by downregulating MMP‐9, thereby promoting wound healing (Guo et al. 2022). LDH and CK are hallmark markers of muscle injury. Overactivation of the MAPK pathway may induce apoptosis or necrosis of muscle cells, disrupting the structure of the cell membrane and leading to the release of intracellular CK and LDH into the bloodstream. Meamarbashi and Abedini (2011) found that POL extract significantly reduced LDH levels at 24 and 48 h post‐exercise and CK levels at 48 h. Asmaliani et al. (2024) determined that POL tea intervention prevented the formation of vascular lesions in the joints of rheumatoid arthritis rats and inhibited joint erosion. Elharrif et al. (2024) administered POL extracts to rat arthritis model and found that it effectively inhibited inflammatory responses and autoimmune processes while positively regulating lipid abnormalities. The combined inhibition of NF‐κβ and MAPK pathways further decreases the release of pro‐inflammatory cytokines. It prevents tissue damage mediated by these cytokines, showcasing the overall advantage of POL in multi‐pathway regulation.

Oxidative stress is a key driver of inflammation, and the Nrf2 signaling pathway plays a critical role in alleviating oxidative stress. Nrf2 is a transcription factor that regulates the expression of antioxidant enzymes such as SOD, CAT, and GPx, effectively reducing ROS production and mitigating oxidative damage to tissues. Kang et al. (2021) determined that HM‐chromanone, extracted from POL, regulates the antioxidant system. It inhibited inflammatory responses and reduced levels of ROS, MDA, nitric oxide (NO), and PGE2 in a dose‐dependent manner. Additionally, Kim et al. (2018) found through cellular experiments that the ethanol extract of POL effectively eliminated free radicals, inhibited NO and ROS production, and suppressed proinflammatory cytokine expression.

### 4.3 POL in Muscle Function Regulation

#### 4.3.1 Skeletal Muscle Relaxation

Skeletal muscles are vital for maintaining body movement, metabolic balance, and life processes. Muscle tension or spasms are often associated with various pathological conditions, such as chronic pain, inflammatory diseases, and exercise‐related injuries. Relaxing excessively tense skeletal muscles not only helps relieve pain and reduce damage but also optimizes metabolic function, supporting muscle repair and overall health. POL and its active ingredients have a certain effect on regulating skeletal muscle (Table 3). Parry et al. found that POL extracts significantly relaxed skeletal muscles and effectively relieved muscle tension in patients with spasms (Parry et al. 1987a). The extracts work quickly and locally, and they may have important clinical applications (Parry et al. 1987b). This mechanism of action may be related to interference with Ca^2+^ (Parry et al. 1993). Okwuasaba et al. determined that POL extracts displayed a skeletal muscle relaxant effect comparable to dantrolene sodium. The mechanism of action appeared to involve the inhibition of transmembrane calcium influx and interference with calcium‐induced calcium release processes (Okwuasaba, Ejike, and Parry 1987a). Further studies revealed that POL extracts increased twitch tension prior to muscle relaxation, which is not a prerequisite for twitch inhibition and occurred independently of electrical stimulation (Okwuasaba et al. 1986; Okwuasaba, Ejike, and Parry 1987b). Additionally, POL extracts exhibited skeletal muscle relaxant effects unique to POL extracts that did not involve cholinergic receptors. These effects were potentially achieved by interfering with Ca^2+^ mobilization in skeletal muscle (Okwuasaba, Parry, and Ejike 1987). The muscle‐relaxing effects of POL have been demonstrated in multiple studies (Habtemariam et al. 1993). Oluwole and Oyedeji (2007) found that POL's relaxing effect on guinea pig ileal smooth muscle may depend on calcium ion antagonism. It could also reduce the intestinal motility index in mice in a dose‐dependent manner. Lu et al. (2009) also observed that POL extract significantly enhanced maximum swimming capacity in mice by improving fat utilization and delaying the accumulation of plasma lactate and ammonia.

**TABLE 3:** Studies on POL's effects in skeletal muscle.

Compound	Dose	Exposure condition	Model	Results	References
(E)‐5‐hydroxy‐7‐methoxy‐3‐(2′‐hydroxybenzyl)‐4‐chromanone (HM‐chromanone) isolated from POL	15 and 30 μM	Cultivate for 24 h	The insulin resistance model was established by treating L6 skeletal muscle cells with 0.75 mM palmitate for 16 h.	Inhibited production of inflammatory cytokines (TNF‐α, IL‐6, IL‐1β, MCP‐1); decreased JNK phosphorylation; blocked IKK, Iκβα, NF‐κβp65 activation; inhibited IRS‐1 serine 307 phosphorylation; increased IRS‐1 tyrosine 612 phosphorylation	Park and Han (2022a)
Mucoadhesive biodegradable device D‐SBD.5HCF (containing purslane leaf extract)	3 mg/cm^2^	A 5 mm × 20 mm film is placed inside the upper lip for 5 or 7 days and administered overnight	Human dermal fibroblast and clinical trial (3 volunteers)	Relaxing muscles, inhibiting the protein levels of MMP1 and MMP3 in fibroblasts, and promoting cell proliferation	Bojanowski et al. (2021)
Aqueous extract of POL	200, 400, 600 mg/kg	Single oral dose	Induced by maximal electroshock and pentylentetrazol in albino mice	Anticonvulsant effects and seizure duration exhibited a marked decrease	Devi et al. (2016)
Aqueous extract of POL	1 × 10^−6^–3 × 10^−2^ g/mL	In vitro experiments used the method of exposing isolated guinea pig ileum strips to drugs in an organ bath; in vivo experiments were performed by intraperitoneal injection	In vitro experiments primarily involved isolated guinea pig ileum strips, while in vivo experiments entailed overnight fasting of mice for preparation	Smooth muscle relaxation, tension inhibition	Oluwole and Oyedeji (2007)
The effects of aqueous (AEE), dialysable (DIF) and methanol (MEE) extracts of POL stems and leaves	AEE 1.5–5.5 × 10^−3^ g/mL, DIF 0.82–2.5 × 10^−3^ g/mL, MEE 1.03–3.53 × 10^−3^ g/mL	Adding to the physiological solution in the organic bath 40–90 min	Isolated tissue models were prepared using rat phrenic nerve—hemidiaphragm and frog rectus abdominis muscles	Inhibited Twitch tension and tonic tension caused by nerve and direct electrical stimulation, weakened contraction induced by K^+^ and caffeine, and reduced muscle contraction	Okwuasaba, Ejike, and Parry (1987a)
(E)‐5‐hydroxy‐7‐methoxy‐3‐(2′‐hydroxybenzyl)‐4‐chromanone (HM‐chromanone) isolated from POL	1–30 μM	Cultivate for 24 h	L6 skeletal muscle cells	Promoted glucose uptake (GLUT4), induced phosphorylation of IRS‐1 Tyr612, AKT Ser473, and PI3K activation, and stimulated phosphorylation of AMPK Thr172, AS160 Thr642, TBC1D1 Ser237, and ACC through the CaMKK β pathway	Park et al. (2021)
(E)‐5‐hydroxy‐7‐methoxy‐3‐(2′‐hydroxybenzyl)‐4‐chromanone (HM‐chromanone) isolated from POL	10, 25, 50 μM	Cultivate for 24 h	L6 skeletal muscle cells	Downregulated the phosphorylation of PTP1B, JNK, and IKKβ, promoted tyrosine phosphorylation of IRS‐1 and inhibited its serine phosphorylation, activated the PI3K/AKT pathway, promoted GLUT4 translocation to the cell membrane, and increased glycogen synthesis	Park and Han (2022b)
Methanol, diethyl‐ether, aqueous extracts of POL	0.82–5.50 mg/mL	In vitro experiments were conducted by incubating in a physiological solution. In vivo experiments were conducted by intravenous injection	Phrenic nerve‐hemidiaphragm and frog muscle	Twitch amplitude initially, followed by sustained relaxation	Okwuasaba, Ejike, and Parry (1987b)
Ethanolic extract of POL	200, 400 mg/kg (in vivo); 1, 2, 5 mg/mL (in vitro)	Single, intraperitoneal injection	Convulsion mice model and anti‐nociceptive activity rat model	Reduced spontaneous activity in mice, prolonged seizure time, and enhanced the analgesic effect while inhibiting muscle contraction	Radhakrishnan et al. (2001)
POL extract	25, 50, 100 μg/mL	Cultivate for 24 h	3 T3‐L1 adipocytes	Promoted glucose uptake and enhanced the phosphorylation of IRS‐1, PI3K, AKT, and AMPK, as well as increasing GLUT4 expression on the cell membrane	Park et al. (2018)
POL extract	400 mg/kg	6 weeks, oral	Type 2 diabetes mice model	Blood glucose, glycated hemoglobin, insulin levels, and HOMA‐IR were significantly reduced, while serum total cholesterol and triglyceride levels decreased. At the same time, the PI3K/AKT and AMPK pathways were activated in skeletal muscle, increasing the expression of PM‐GLUT4	Lee et al. (2020)
Aqueous extract of POL	70–140 mg	Single topical application on affected muscle, 2 h	Patients with muscle spasticity	Effective spasticity relief	Parry et al. (1987a)
Aqueous extract of POL	200–1000 mg/kg (intraperitoneal injection), 5 g/kg (oral administration)	Single oral dose or intraperitoneal injection	Normal adult Wistar rats and 1‐month‐old BALB/c mice	Significant muscle relaxation effect	Parry et al. (1987b)
Ethanolic and aqueous extract of POL	10%, 20%, 30% (v/v)	Soaked in solution	Chick biventer cervicis muscle preparation	Reduced muscle spasms and spasms	Habtemariam et al. (1993)
Aqueous extract of POL	1, 2, 3, 4 mg/mL	Soaked in solution	The diaphragm tissue was taken and placed in a 50 mL organ bath containing double glucose Tyrode solution	K^+^ concentration in the extract was significantly and positively correlated with the effectiveness of inhibiting diaphragm twitching and contraction	Parry et al. (1993)
Aqueous extract of POL	5–50 × 10^−4^, 5–10 × 10^−3^ g/mL	Contact with the organization for 30–90 min	Rat phrenic nerve‐hemidiaphragm, frog sciatic nerve‐sartorius, and rectus abdominis	Reduced twitch and tonic tension caused by electrical stimulation and weakened K^+^‐induced contraction	Okwuasaba et al. (1986)
Purslane extracts	100, 200, 400 mg/kg	28 days, intraperitoneal injection	Male mice were used for modeling via forced swimming exercise	Plasma triglycerides, lactates, and ammonia levels all decreased, while plasma glucose levels increased	Lu et al. (2009)
Extracts of POL	1.20–8.25 × 10^−3^ g/mL	The extracts were added to the physiological solution of the organ bath containing isolated muscle tissues	Rat phrenic nerve‐hemidiaphragm and frog rectus abdominis	Inhibited twitch tension and contracture	Okwuasaba, Ejike, and Parry (1987b)

Skeletal muscles are key organs in glucose metabolism, and POL can effectively improve skeletal muscle metabolic function by activating multiple signaling pathways. Park and Han (2022a, 2022b) studies indicated that HM‐chromanone activates the adenosine 5′‐monophosphate (AMP)‐activated protein kinase (AMPK) pathway, which inhibits mTOR/S6K1 signaling. This ultimately downregulates the phosphorylation levels of Ser307 and Ser632 of insulin receptor substrate‐1 (IRS‐1) while upregulating the phosphorylation of its key activation site (Tyr612). The combined regulatory effect on the serine/threonine phosphorylation state of IRS‐1 and the inhibitory effect on negative regulators of insulin signaling and inflammatory activated protein kinases block palmitate‐induced dysfunction of the insulin signaling pathway, effectively improving insulin resistance in L6 skeletal muscle cells. The research team found that HM‐chromanone stimulates glucose uptake in L6 skeletal muscle cells by activating the PI3K/AKT and CaMKKβ/AMPK pathways (Park et al. 2021). It also promoted glycogen synthesis through the GSK3α/β pathway. POL extract promoted glucose uptake in 3T3‐L1 adipocytes by activating the PI3K/AKT and AMPK pathways, thereby enhancing glucose transporter 4 (GLUT4) transport to the cell membrane (Park et al. 2018). Lee et al. (2020) concluded that POL extract enhanced insulin resistance in mouse skeletal muscle by activating the PI3K/AKT and AMPK pathways while relaxing the muscle.

Bojanowski et al. (2021) invented a patch containing POL extract that effectively expands gingival coverage. The formulation's components positively affected fibroblasts in vitro. This delivery platform may benefit both the skin and the oral cavity. Devi et al. (2016) discovered that POL leaf extract exhibited significant anticonvulsant properties in mice, which may be attributed to enhanced GABAergic transmission in the central nervous system. Radhakrishnan et al. (2001) confirmed that POL extracts could prolong pentylenetetrazole‐induced seizure duration and exhibit in vitro and in vivo muscle relaxant activity. The in vitro effect may be related to high potassium concentrations.

#### 4.3.2 Smooth Muscle Relaxation

Smooth muscle is essential for regulating airway, gastrointestinal, and blood vessel functions. Abnormal activity of smooth muscle could lead to conditions such as asthma, chronic obstructive pulmonary disease, and other related disorders. Effective regulation of smooth muscle could reduce symptoms and improve quality of life. POL and its active ingredients have a certain effect on regulating smooth muscle (Table 4). Boroushaki et al. (2004) conducted a study in which they administered boiled purslane extract to guinea pigs. They found that the number of lemon‐induced coughs in the purslane group was significantly lower than in the saline group, indicating that purslane can effectively relax smooth muscles. Malek et al. (2004) further determined that boiled purslane extract improved lung function parameters, including forced expiratory volume in 1 s (FEV_1_), peak expiratory flow (PEF), maximum mid‐expiratory flow (MEF_25–75_), and specific airway conductance (sGaw), in patients with asthma. Parry et al. (1988) found that purslane extract dose‐dependently relaxes smooth muscle, contracts the aorta, inhibits atrial function, and increases blood pressure in rats. Boskabady et al. (2016) revealed that purslane extract stimulates β₂‐adrenergic receptors in tracheal smooth muscle and may inhibit histamine H₁ receptors. They also found that water‐ethanol extracts of purslane significantly ameliorate asthma in rats by improving pulmonary inflammation and oxidative stress through anti‐inflammatory and antioxidant actions (Boskabady et al. 2019).

**TABLE 4:** Studies on POL's effects in smooth muscle.

Compound	Dose	Exposure condition	Model	Results	References
POL aqueous boiled extract	2.5% w/v, 5% w/v	Aerosol exposure 10 min before citric acid‐induced cough	Guinea pigs exposed to 0.1 g/mL citric acid aerosol for 7 min	Reduced cough counts have been demonstrated to be as efficacious as codeine	Boroushaki et al. (2004)
POL 5% boiled extract	0.25 mL/kg	Oral administration; pulmonary function measured at 15, 30, 60, 90, 120 min post‐administration	Asthmatic patients	Significant increases in FEV_1_, PEF, MEF_25–75_, and sGaw compared to baseline (similar to theophylline and salbutamol)	Malek et al. (2004)
POL aqueous extract	2.0 × 10^−5^–3.1 × 10^−3^ g/mL (ex vivo tissue); 1.4–56 mg/kg (intravenous drip); 2.8 mg/min (Intravenous infusion)	In vitro, 2–4 min for maximum smooth muscle relaxation, 5–7 min for aortic contraction, and 10–25 min for atrial inhibition; in vivo, pressor effect lasts 1–2 min after intravenous bolus, and 26 min post 30‐min intravenous infusion (until animal death)	Guinea pig fundus/taenia coli; rabbit jejunum/aorta/atria; rat blood pressure	Dose‐dependent relaxation of gut smooth muscle, contraction of rabbit aorta, negative inotropic/chronotropic effects on atria, and pressor responses on rat blood pressure	Parry et al. (1988)
Hydro—ethanolic extract of POL	0.06, 0.12 and 0.25 mg/mL	Exposing the transportation chains to the solution containing the extract for 7 min	Guinea pig tracheal chains were pre ‐ contracted with 10 μM methacholine hydrochloride	Increased β‐adrenoceptor sensitivity: leftward shift of isoprenaline concentration‐response curve; reduced EC_50_ (dose ratio‐1) compared to control	Boskabady et al. (2016)
Ethanolic extract of Portulaca oleracea	1, 2, and 4 mg/mL	21 days, oral	Sensitized rats	Concentration‐dependent reduction in bronchoalveolar lavage fluid (BALF) levels of total protein (TP), phospholipase A_2_ (PLA_2_), and immunoglobulin E (IgE) compared to sensitized controls	Kaveh et al. (2017)
Hydro‐ethanol extract from the aerial part of POL	0.25, 0.50 and 1.00 mg/mL	Exposing tracheal smooth muscle to solutions containing the extract for 10 min	Guinea pig tracheal chains were used for the experiment, and the tissues were equilibrated for at least 1 h	Accelerated sciatic nerve conduction velocity, reduced tail‐flick latency, improved hyperglycemia/oxidative stress, upregulated NGF‐β, and downregulated TNF‐α/IL‐6	Hashemzehi et al. (2016)
Hydro—ethanolic extract of POL and its constituent ALA	0.2 and 0.4 mg/mL	21 days, oral	Sensitized rats	Reduced tracheal responsiveness to methacholine/ovalbumin; decreased BALF levels of TP, PLA_2_, IgE, IL‐4; increased INF‐γ and INF‐γ/IL‐4 ratio (restored Th1/Th2 balance)	Kaveh et al. (2019)
Hydro—ethanolic extract of POL	40, 80, 160 mg	21 days, oral	Asthmatic rats	Reduced total WBC/neutrophil/eosinophil/monocyte counts, NO_2_/NO_3_/MDA levels, and lung pathology (fibrosis/emphysema/inflammation); increased lymphocyte count, SOD/CAT/thiol levels (oxidative biomarkers)	Boskabady et al. (2019)
POL leaf extract	5 and 50 mg/kg	Intragastric administration for 15 days	ICR mice	Significantly enhanced macrophage phagocytic activity and splenic lymphocyte proliferation ability, and reduced intestinal motility in mice	Catap et al. (2018)

Similarly, Kaveh et al. (2017) found that purslane extracts exhibit anti‐inflammatory and immunomodulatory effects in sensitized rats, comparable to or more pronounced than those of dexamethasone. Their research indicated that purslane water‐ethanol extracts and their component α‐linolenic acid (ALA) can increase interferon‐γ levels and the IFN‐γ/interleukin‐4 ratio (Kaveh et al. 2019). Hashemzehi et al. (2016) also found that purslane extracts inhibit muscarinic receptors in guinea pig tracheal smooth muscle, particularly blocking H₁ receptors. Catap et al. (2018) found that ethyl acetate extracts from POL leaves induced more significant intestinal motility slowing, and that ethanol extracts had effects comparable to atropine.

#### 4.3.3 Cardiac Muscle Relaxation

Damage or dysfunction of myocardial cells is a major pathological basis for many cardiovascular diseases (Table 5). In terms of antioxidant mechanisms, POL extract plays a significant role in reducing oxidative stress‐induced damage to myocardial tissue. Khodadadi et al. (2018) revealed that POL seed water‐ethanol extract increased body weight and total thiol levels in rats with subclinical hyperthyroidism, while reducing left ventricular development pressure (LVDP) and MDA levels. The study revealed that POL has a protective effect against thyroid hormone‐induced cardiac dysfunction. Its effects on increasing body weight and lowering blood pressure are superior to those of vitamin C. Pakdel et al. (2022) found that POL seed water‐ethanol extract reduced heart rate, systolic blood pressure, free T_4_ levels, MDA, and nitric oxide metabolite levels in thyrotoxicosis rats while increasing thiol concentration and the activities of superoxide dismutase and catalase. Ou et al. (2007) also found that POL water extract reduced MDA content in the myocardial mitochondria of a D‐galactose‐induced aging model in mice. It increased the relative content of cardiolipin and the activities of Ca^2+^‐ATPase, complex I, and complex II + III. POL exerts its protective effect on mitochondria by inhibiting the lipid peroxidation of myocardial mitochondrial phospholipids and improving respiratory chain enzyme activity. Bidkar et al. (2011) used fresh POL juice to study isolated frog hearts and found that POL produced a positive inotropic effect.

**TABLE 5:** Studies on POL's effects in cardiac muscle.

Compound	Dose	Exposure condition	Model	Results	References
POL fresh aerial part juice	0.1,0.2, 0.3 mL	Venous cannula	Isolated frog heart	Reduced heart rate, increased force of contraction	Bidkar et al. (2011)
POL seed hydro‐alcoholic extract	400 mg/kg	2 weeks, intraperitoneal injection	Rats with subclinical hyperthyroidism	Lower heart rate and systolic blood pressure；improved oxidative stress (reduced MDA/NO metabolite, increased thiol/SOD/CAT)	Pakdel et al. (2022)
POL seeds hydro‐alcoholic extract	100, 200, 400 mg/kg	Drinking water for 4 weeks	Rats with subclinical hyperthyroidism	Increased body weight, decreased left ventricular development pressure (LVDP), decreased MDA levels, and increased total sulfhydryl levels in rats	Khodadadi et al. (2018)
POL water extract	13 g/kg	30 days, intraperitoneal injection	D‐galactose‐induced aging mice model	Decreased myocardial mitochondria lipid peroxidation (MDA); improved respiratory chain enzyme activity (Complex I and II + III)	Ou et al. (2007)

### 4.4 Balancing Bone Immunity

Bone immunity represents the complex interaction between the skeletal and immune systems, playing an indispensable role in maintaining skeletal health and immune balance. Bone marrow is not only crucial for hematopoiesis and immune cell generation but also serves as a core regulator of systemic immune function. POL has shown significant effects in improving bone marrow hematopoietic function and immune activity (Table 6). In studies on immune suppression from cyclophosphamide, POL polysaccharides increased the size of the spleen and thymus. They also improved the activity of NK cells and LAK cells (Meng et al. 2017). These actions are mediated by modulation of the microenvironment of bone marrow stromal cells and hematopoietic stem cells, resulting in increased IL‐2 and IFN‐γ secretion and significant inhibition of inflammatory factors such as TNF‐α and IL‐6. The regulation of the NF‐κβ pathway further stabilizes the bone marrow immune microenvironment, protecting skeletal health. Moreover, POL indirectly influences the bone immune system by modulating intestinal immune function. Research indicates that it significantly stimulates macrophage, dendritic cell, and T‐cell activity in Peyer's patches, enhancing intestinal immune barrier function (Georgiev et al. 2017). This localized immune enhancement not only reduced the release of systemic inflammatory factors but also optimized the signaling communication between bone marrow and the intestines via the bone marrow–intestine axis.

**TABLE 6:** Studies on POL in balancing bone immunity.

Compound	Dose	Exposure condition	Model	Results	References
Portulaca complex extract	1080 mg/day	8 weeks, oral	Healthy individuals	NK cell activity significantly increased, and IFN‐γ and IL‐12 serum concentrations significantly increased	Jung et al. (2024)
POL polysaccharide	180, 90 mg/kg	10 days, intraperitoneal injection	Immunosuppressed mouse model	Improved spleen index and thymus index, enhanced NK cell and LAK cell activity, and increased serum IL‐2, IL‐4, IL‐10, and IFN‐γ	Meng et al. (2017)
Hydro‐ethanolic extract of POL	10, 40 160 μg/mL	Cultivate for 72 h	Isolated human lymphocytes	Decreased cell proliferation, NO production, and secretion of IL‐4/IL‐10/IFN‐γ; significantly enhanced Th1/Th2 (IFN‐γ/IL‐4) and Treg/Th2 (IL‐10/IL‐4) balances	Askari et al. (2016)
Acidic polysaccharide complexes from purslane	100 μg/mL	Cultivate for 48 h	Peyer's patch cells from normal mice and human peripheral blood leukocyte	Promoted the activity of human blood T cell subsets (CD4+/CD25+ and CD8+/CD25+) as well as phagocytes (CD14+ and CD64+), induced IL‐6 production and enhanced ROS generation	Georgiev et al. (2017)
Ethanol extract of POL	10 and 100 μg/mL	Cultivate for 24 h	Peripheral blood mononuclear cells and head kidney leukocytes of striped catfish	Significantly increased lysozyme activity and total immunoglobulin levels	Nhu et al. (2019)

In regulating immune system balance, POL exerts unique effects by influencing Th1/Th2 immune responses. Jung et al. (2024) research revealed that POL and perilla mixed extracts significantly increased Th1‐type cytokine levels, including IFN‐γ and IL‐12, and enhanced NK cell activity. IL‐12 is a key cytokine that promotes Th1 cell differentiation. Its elevated levels induce additional Th1‐type immune responses. As a hallmark cytokine of Th1 cells, IFN‐γ activates macrophages, enhances NK cell cytotoxicity, and strengthens cellular immune function. Askari et al. (2016) also demonstrated that POL ethanol extract significantly reduced the proliferation rate and the production and secretion of NO and IL‐4, IL‐10, and IFN‐γ in human lymphocytes stimulated via PHA. Meanwhile, it significantly improved the Th1/Th2 (IFN‐γ/IL‐4) and Treg/Th2 (IL‐10/IL‐4) balance. Nhu et al. (2019) showed that POL ethanol extract could really boost the lysozyme activity, complement activity, and total immunoglobulin levels in peripheral blood mononuclear cells of striped catfish.

### 4.5 Regulating Bone Metabolism

Bone metabolism is a vital physiological process for maintaining skeletal stability and systemic mineral balance, regulated by the dynamic balance between osteoblasts and osteoclasts. Osteoblasts are responsible for new bone formation, while osteoclasts resorb bone tissue to complete bone remodeling. These cells maintain a relative balance under healthy conditions. Still, when this balance is disrupted—such as through insufficient osteoblast activity or excessive osteoclast activity—it may lead to metabolic bone diseases such as osteoporosis, severely affecting quality of life. Therefore, the development of intervention strategies based on natural compounds to restore bone metabolism balance has become an important research focus in recent years. POL, a traditional medicinal and edible plant, has gained attention in bone metabolism research due to its rich active components and significant multi‐target regulatory effects (Table 7).

**TABLE 7:** Studies on POL in regulating bone metabolism.

Compound	Dose	Exposure condition	Model	Results	References
Viscozyme‐assisted polysaccharide	In vitro experiments at 12.5–100 μg/mL, in vivo experiments at 25, 50, and 100 μg/mL	Cultivate for 24 h for in vitro experiments and 4 days for in vivo experiments	MC3 T3‐E1 Cells, Zebrafish	Reduced ROS levels and cell apoptosis rate, upregulated Nrf2 and Keap1 expression, downregulated HO‐1 and NQO1 expression, and promoted bone growth	Fu et al. (2022)
POL ethanol extract	25, 50, 100 μg/mL	Cultivate for 2–4 days	Bone marrow derived macrophages	Inhibited RANKL‐induced intracellular Ca^2+^ oscillations and NFATc1 activation, while reduced RANKL‐mediated cytotoxicity and PARP cleavage, ultimately promoted TRAP^+^ multinucleated cell formation and elevated TRAP activity	Erkhembaatar et al. (2015)
Silicon‐biofortified purslane	111‐206 μg/L	Cultivate for 36 h	Caco‐2 cell model, human osteoblast cells	In human osteoblasts, alkaline phosphatase mRNA levels were increased by approximately 1.4–4.3 times and type I collagen mRNA levels were increased by approximately 1.6–5.6 times	D'Imperio et al. (2017)
Ethanol extract of purslane	In vitro experiments (10, 25, 50 μg/mL), in vivo experiments (250 mg/kg)	Cultivate for 4 days (vitro experiment); intraperitoneal injection for 8 days (vivo experiment)	LPS‐induced osteolysis mouse model, osteoclast differentiation model	Inhibited the phosphorylation of AKT and GSK3β, downregulated the expression of c‐Fos, NFATc1, and osteoclast‐specific genes (TRAP, OSCAR, DC‐STAMP), disrupted the actin ring structure, and reduced bone resorption pits, without cytotoxicity; In vivo, it improved LPS‐induced bone loss, increased BV/TV and Tb.N, decreased Tb.Sp, reduced bone erosion, and decreased the number of TRAP‐positive osteoclasts	Kim et al. (2015)
Viscozyme‐assisted polysaccharide	25, 50, 100 μg/mL	Cultivate for 7–9 days	Zebrafish osteoporosis model	Reduced ROS levels, restored bone mineralization, inhibited cell apoptosis (elevated Bcl‐2 levels, reduced levels of caspase‐3, Bax, and cytochrome C), and gradually restored 28 potential biomarkers in zebrafish, regulating metabolic pathways such as arachidonic acid and tyrosine	Yue et al. (2024)
POL extracts	15 and 45 mg/kg bw/day	4 weeks, oral	Nutrient intake imbalance rat model	Enhanced the IGF‐1 signaling pathway in osteoblasts, activated the TGF‐β signaling pathway by increasing the expression of BMP‐2 and Smad4, promoted cell proliferation, reduced the levels of bone turnover markers in serum, increased bone density, improved glucose metabolism and insulin resistance, and reduced fat mass	Ko et al. (2019)
Mori Follum, POL, *Lycium barbarum* L.	10, 50, 100, 200, 500 μM	Cultivate for 24 h、7 days、14–21 days	MC3T3‐E1 cells	Promoted the proliferation, differentiation, and mineralization of MC3 T3‐E1 cells, and upregulated the expression of key proteins in the PI3K/AKT/Runx2 pathway	Jia et al. (2024)
POL extract	250 mg/kg	Once every 2 days, lasting for 8 days, orally administered	Osteolysis mice model	Bone loss and bone erosion occurred, and the number of TRAP‐positive osteoclasts decreased, while bone volume/total volume (BV/TV) and trabecular number (Tb.N) increased, and trabecular separation (Tb.Sp) improved	Shen et al. (2022)
Ethanolic purslane extract	100 mg/kg	Intraperitoneal injection for 8 weeks	Glucocorticoid‐induced osteoporosis model in rats	Improved dexamethasone‐induced decreases in bone density and bone mineral content, regulated bone metabolism markers (reduced ALP and ACP activities, increased OCN levels), inhibited inflammatory factors (TNF‐α, IL‐6), and activated antioxidant pathways (Nrf2/HO‐1)	Saleh et al. (2024)

Shen et al. (2022) research found that purslane intervention reduced osteoclast differentiation and bone resorption activity. This occurred by inhibiting the AKT/GSK3β‐c‐Fos‐NFATc1 signaling pathway. As a result, bone loss and erosion were reduced. Additionally, Kim et al. (2015) revealed that purslane impeded RANKL‐induced osteoclast differentiation and suppressed actin ring formation by obstructing the AKT/GSK3β‐c‐Fos‐NFATc1 signaling pathway. Erkhembaatar et al. (2015) conducted experiments with purslane ethanol extracts and discovered that these extracts could reduce Ca^2+^ oscillations and NFATc1 amplification. This occurs by inhibiting RANKL‐induced intracellular Ca^2+^ storage release and weakening RANKL‐mediated cytotoxicity. Ultimately, this enhances RANKL‐mediated osteoclast generation. D'Imperio et al. (2017) found that purslane increased the expression of two osteoblast markers, type I collagen and alkaline phosphatase (ALP), in human osteoblasts.

Numerous studies have found that POL could regulate bone metabolism by modulating oxidative stress. Jia et al. (2024) research utilized an extract composed of purslane, mulberry branches, and goji berries, which was found to promote the proliferation, differentiation, and mineralization of MC3T3‐E1 cells through the PI3K/AKT/Runx2 signaling pathway. Furthermore, the study demonstrated an increase in the expression of PI3K, AKT, their phosphorylated proteins, and Runx2. Fu et al. (2022) confirmed that the polysaccharide extract of purslane regulates the Nrf2/Keap1 pathway, increasing the expression levels of HO‐1, NQO1, Nrf2, and Keap1, while reducing cell apoptosis and ROS levels. This results in a reduction of ROS production and lipid peroxidation, thereby promoting bone growth and exhibiting anti‐osteoporosis effects. The fourth item is hereby indicated. Saleh et al. (2024) further analyzed that purslane ethanol extracts can increase GSH content and enhance the antioxidant capacity of SOD, GPx, and GST activity, thereby regulating bone metabolism‐related indicators such as OPG/RANKL. This upregulates the expression of Nrf2/HO‐1 pathway proteins, downregulates the expression of p‐IKK and NFATc1 proteins, improves the bone microstructure of osteoporotic rats, and exerts an anti‐osteoporotic effect.

In the study, Ko et al. (2019) observed that purslane intervention led to several notable outcomes. These include an increase in the length of the femur and tibia, a reduction in serum ALP and osteocalcin levels, an improvement in bone mineral density, a decrease in leg fat mass, an enhancement of insulin resistance, and a promotion of osteoblast proliferation through the activation of the TGF‐β and IGF‐1 signaling pathways. Yue et al. (2024) research revealed that the polysaccharides found in purslane can reduce the levels of certain proteins, including caspase‐3, Bax, and cytochrome C. This reduction occurs through the regulation of the mitochondrial‐dependent pathway, which in turn inhibits the process of apoptosis. Furthermore, POL has been observed to progressively restore 28 potential biomarkers to their normal levels in zebrafish. In addition, POL has been shown to intervene in the metabolic pathways of arachidonic acid, tyrosine, phenylalanine, and sphingolipids, thereby exerting an anti‐osteoporosis effect.

## 5 Drug Toxicity and Safety

Although POL exhibits numerous remarkable pharmacological properties, potential toxicity and safety concerns have gradually gained attention, particularly with long‐term or excessive use. While components such as α‐linolenic acid and polyphenols present in POL offer significant health benefits, excessive intake may pose health risks. Studies suggest that POL may have certain negative effects on the male reproductive system (Alipour et al. 2022). In animal experiments using high doses of POL extract, male rats exhibited a significant reduction in sperm count and decreased sperm motility. They observed blood stasis and cellular damage in their testes and epididymis. Although low doses of POL may provide some protection for reproductive health, excessive use may lead to fertility‐related issues (Farag et al. 2021). Furthermore, excessive use of POL may have adverse effects on the gastrointestinal system. The oxalates in POL can bind with calcium in the body to form calcium oxalate crystals, which may accumulate in the kidneys and lead to conditions such as kidney stones. Prolonged or excessive consumption of POL could result in gastrointestinal discomfort, including symptoms such as nausea, vomiting, and diarrhea, all related to the accumulation of oxalates and their reaction products (Okafor et al. 2021). Due to the limited research on the toxic mechanisms and long‐term safety of POL, further studies are needed. In particular, the impact on various physiological systems requires more in‐depth evaluation to assess the long‐term use and potential side effects, ensuring its positive role in daily use (Petropoulos et al. 2019; YouGuo et al. 2009).

## 6 Conclusion

With the global aging population, the prevalence of musculoskeletal disorders such as sarcopenia, osteoarthritis, and osteoporosis is rising annually, becoming a worldwide issue that affects health and quality of life. As a food medicine homologous plant, POL, with its rich nutritional and bioactive components, shows great promise in the treatment of musculoskeletal disorders. This study summarizes the specific mechanisms of action of POL in musculoskeletal disorders. POL and its active components can significantly increase the activity of antioxidant enzymes by activating the Nrf2/ARE pathway. Additionally, POL inhibits the NF‐κβ pathway, reducing the production of inflammatory factors and alleviating tissue damage caused by inflammation. Moreover, POL modulates the MAPK signaling pathway, inhibiting the activity of kinases such as ERK, JNK, and p38, further reducing skeletal and muscle damage induced by inflammation, demonstrating its dual anti‐inflammatory and tissue‐protective effects. In improving muscle function, POL activates the PI3K/AKT pathway to enhance glucose uptake by muscles, improve insulin sensitivity, and promote muscle metabolism and energy utilization. Furthermore, POL stimulates the PI3K/AKT pathway to promote the proliferation and differentiation of osteoblasts, enhancing the synthesis and mineralization of bone matrix, thus improving bone density and strength.

Despite the significant potential of POL in treating musculoskeletal disorders, current research still has certain limitations. The pharmacological mechanisms of POL are complex. Although it contains various active components, different extraction methods and dosages may affect its bioavailability and therapeutic efficacy. While animal models and in vitro experiments support the therapeutic effects of POL, there is a lack of large‐scale clinical trial data, particularly regarding long‐term efficacy and safety. Future clinical trials will provide a more solid scientific basis for its widespread application. The active components of POL may exhibit either synergistic or antagonistic effects, which present higher demands for its clinical use. Furthermore, research on POL's pharmacokinetics, especially in terms of absorption, metabolism, and distribution, is still relatively weak. More detailed studies are needed to reveal its metabolic pathways and bioavailability within the body. Future research should explore its therapeutic effects under different clinical conditions, optimize treatment protocols, and validate its efficacy and safety through more clinical trial data.

## Author Contributions

**Yanxin Li:** methodology (equal), writing – original draft (lead), writing – review and editing (equal). **Yan Wang:** conceptualization (supporting), writing – original draft (supporting), writing – review and editing (equal). **Xiaotian Feng:** data curation (equal), writing – review and editing (equal). **Qiyu Wang:** data curation (equal), writing – review and editing (equal). **Hongwei Cui:** visualization (equal), writing – review and editing (equal). **Tiancheng Ma:** visualization (equal), writing – review and editing (equal). **Yujing Chen:** data curation (equal), writing – review and editing (equal). **Jiajun Liu:** data curation (equal), writing – review and editing (equal). **Jianxiong Ma:** conceptualization (equal), writing – review and editing (supporting).

## Conflicts of Interest

The authors declare no conflicts of interest.

## Data Availability

All datasets supporting the conclusions of this study were obtained from public databases and have been described in this article.
